# Job demands, job resources, and burnout among metro site management personnel in China: a cross-sectional study based on the JD-R model

**DOI:** 10.3389/fpsyg.2026.1856402

**Published:** 2026-07-23

**Authors:** Chunxiao Zhao, Jinghua Qi, Yuxiang Chen

**Affiliations:** 1 State Key Laboratory of Intelligent Construction and Healthy Operation and Maintenance of Deep Underground Engineering, China University of Mining and Technology, Xuzhou, China; 2 School of Mechanics and Civil Engineering, China University of Mining and Technology, Xuzhou, China

**Keywords:** burnout, China, JD-R model, job demands, job resources, metro projects, occupational health, site management personnel

## Abstract

**Introduction:**

Burnout has been widely examined in healthcare, education, and public-sector occupations, but less is known about employees working in high-risk, project-based environments. Drawing on the Job Demands-Resources (JD-R) model, this study examined the relationships of job demands and job resources with burnout among metro site management personnel in China.

**Methods:**

A two-stage cross-sectional survey was conducted. A pilot survey with 140 valid responses was used to refine the measurement scales, followed by a formal survey with 274 valid responses. Burnout was assessed through exhaustion, cynicism, and low professional efficacy. Reliability, validity, correlation, and hierarchical regression analyses were conducted.

**Results:**

Exhaustion (M = 4.502) and cynicism (M = 4.237) were more pronounced than low professional efficacy (M = 3.638). Job demands were positively associated with burnout, whereas job resources were negatively associated with burnout. Workload showed the largest standardized coefficient among the separate demand-side regression models (β = 0.608). Social support, distributive justice, and career development opportunities showed the strongest negative associations among the resource-side models.

**Discussion:**

The findings support the applicability of the JD-R framework in high-risk, project-based metro construction settings and highlight workload management and organizational resources as practical priorities. Because the study was cross-sectional and based on self-reported data, the results should be interpreted as associations rather than causal effects.

## Highlights

- Exhaustion and cynicism were relatively more pronounced than low professional efficacy in the formal sample.
- Workload showed the largest standardized coefficient among the demand-side regression models (β = 0.608).
- Social support, distributive justice, and career development opportunities were the most influential resource-side buffers.

## 1 Introduction

Job burnout has become an increasingly important topic in organizational and occupational research because it undermines employee wellbeing, job performance, and organizational effectiveness (Freudenberger, 1974; Maslach et al., 2001). While substantial evidence has accumulated in healthcare, education, policing, and public administration, much less attention has been paid to burnout in project-based work settings, especially in temporary organizations characterized by schedule pressure, environmental uncertainty, and coordination complexity.

This omission is noteworthy in infrastructure construction. Metro projects are large-scale, technically complex, and operationally risky systems. They often involve underground work, tight schedules, multiple stakeholders, and difficult construction interfaces (Ding and Xu, 2017). In such settings, site management personnel work under sustained pressure while simultaneously handling safety responsibility, production coordination, and organizational communication. Their psychological condition can therefore influence not only personal wellbeing, but also coordination quality and project execution.

The Job Demands-Resources model offers a strong analytical lens for understanding burnout in such contexts. The model posits that burnout develops when job demands chronically exceed the resources available to employees (Demerouti et al., 2001; Bakker et al., 2004). The model has been widely applied across occupations and has repeatedly shown explanatory value for burnout, engagement, and performance. However, its application to metro site management personnel remains limited. Previous research has documented burnout among construction project managers in China (Yang et al., 2017), but empirical evidence focused specifically on metro site management personnel remains limited.

Metro site management personnel differ from employees in more stable organizational settings in several important ways. Their work is embedded in temporary project organizations, involves safety-critical underground operations, and requires continuous coordination among contractors, owners, designers, supervisors, government agencies, and front-line teams. In addition, work locations are often unstable, project schedules are compressed, and managers are simultaneously accountable to both the parent company and the project organization. These features may make workload, role conflict, work-family conflict, and job complexity particularly salient job demands, while social support, distributive justice, career development opportunities, and job autonomy may function as especially important resources in this context.

This study does not seek to propose a new version of the JD-R model. Rather, it contextualizes the JD-R framework in a temporary, high-risk, multi-stakeholder infrastructure project setting. Its contribution lies in identifying which demand and resource dimensions are most salient for burnout-related scores among metro site management personnel, an occupational group that has received limited attention in organizational psychology and project management research.

## 2 Materials and methods

### 2.1 Theoretical background and hypotheses

The JD-R model distinguishes between job demands and job resources. Job demands are aspects of work that require sustained physical, cognitive, or emotional effort and therefore carry physiological or psychological costs. Job resources are aspects of work that help employees attain work goals, reduce the cost of demands, or stimulate growth and motivation (Demerouti et al., 2001; Bakker et al., 2004).

In metro project settings, workload arises from compressed schedules, overtime, and large task volume; role conflict stems from inconsistent expectations across the project team and the parent organization; work-family conflict reflects the strain caused by long on-site presence and unstable work locations; and job complexity reflects technical difficulty, coordination load, and changing work interfaces. Prior work suggests that each of these factors can intensify burnout (Leung et al., 2011; Alarcon, 2011; Cao et al., 2020).

On the resource side, social support provides emotional and instrumental help, job autonomy increases perceived control, career development opportunities support long-term motivation, and distributive justice shapes employees’ evaluation of the effort-reward relationship. Existing research has shown that such resources can mitigate strain and reduce burnout (Kirmeyer and Shirom, 1986; Van Yperen and Hagedoorn, 2003; Gabris and Ihrke, 2001). Evidence from safety-critical occupations also suggests that perceived organizational support is associated with lower burnout (Zeng et al., 2020).

**FIGURE 1:**
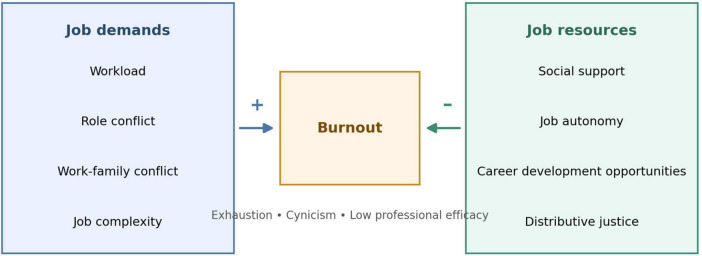
Conceptual research framework based on the JD-R model.

Accordingly, this study proposed two overarching hypotheses: H1, job demands are positively associated with burnout; and H2, job resources are negatively associated with burnout. Dimension-level hypotheses were specified for each demand and resource factor. The conceptual framework is presented in Figure 1. The study variables and directional hypotheses are summarized in Table 1.

**TABLE 1:** Study variables and directional hypotheses.

Domain	Construct	Expected relationship with burnout	Illustrative source
Job demand	Workload	Positive	Voydanoff (2004)
Job demand	Role conflict	Positive	Rizzo et al. (1970)
Job demand	Work-family conflict	Positive	Carlson et al. (2000)
Job demand	Job complexity	Positive	Karasek et al. (1998); Chung-Yan (2010)
Job resource	Social support	Negative	Van Yperen and Hagedoorn (2003)
Job resource	Job autonomy	Negative	Kirmeyer and Shirom (1986)
Job resource	Career development opportunities	Negative	Bakker et al. (2004); Chen and Chen (2012)
Job resource	Distributive justice	Negative	Gabris and Ihrke (2001)

### 2.2 Study design and participants

This study employed a two-stage cross-sectional survey design.

Prior to the formal investigation, a pilot survey was conducted to evaluate and refine the measurement instrument. A total of 163 pilot questionnaires were collected, of which 140 were retained after data screening. Reliability analysis and exploratory factor analysis were performed to assess the psychometric properties of the preliminary scales. Based on statistical performance and expert evaluation, items demonstrating weak psychometric characteristics or conceptual redundancy were removed or revised before the formal survey.

The formal survey yielded 328 returned questionnaires. After excluding incomplete, duplicate, and invalid responses, 274 valid questionnaires remained, resulting in an effective response rate of 83.5%. The demographic characteristics of the formal sample are summarized in Table 2.

**TABLE 2:** Key characteristics of the formal sample (*n* = 274).

Category	Distribution
Gender	Male 224 (81.8%); female 50 (18.2%)
Age	< 25: 39 (14.2%); 25–30: 73 (26.6%); 31–35: 78 (28.5%); 36–45: 54 (19.7%); 46–55: 25 (9.1%); ≥ 56: 5 (1.8%)
Education	Below college: 8 (2.9%); college: 90 (32.8%); undergraduate: 145 (52.9%); graduate and above: 31 (11.3%)
Marital status	Unmarried 77 (28.1%); married 197 (71.9%)
Work tenure	< 5 Years: 59 (21.5%); 5–10: 103 (37.6%); 11–15: 68 (24.8%); 16–20: 19 (6.9%); > 20: 25 (9.1%)
Daily working hours	≤ 8 h: 32 (11.7%); 9–10 h: 114 (41.6%); 11–12 h: 101 (36.9%); > 12 h: 27 (9.9%)
Monthly income	< 5,000 RMB: 13 (4.7%); 5,000–7,000: 92 (33.6%); 7,000–9,000: 72 (26.3%); 9,000–11,000: 34 (12.4%); > 11,000: 63 (23.0%)

Participants were recruited through a combination of online and offline questionnaire distribution channels. Eligible respondents were required to be currently engaged in metro construction site management activities and possess responsibilities related to project coordination, supervision, safety management, technical management, or construction administration.

To reduce response bias, participation was voluntary and anonymous. Prior to questionnaire completion, respondents were informed of the purpose of the study, the confidentiality of the collected information, and their right to discontinue participation at any time.

### 2.3 Measures

The questionnaire was adapted from established scales in burnout and organizational behavior research and further refined to reflect the occupational characteristics of metro site management personnel.

Burnout was measured using a scale derived from the Maslach Burnout Inventory–General Survey (MBI–GS), comprising the dimensions of exhaustion, cynicism, and low professional efficacy (Maslach and Jackson, 1981; Maslach et al., 1996, 2001).

Job demand measures included workload, role conflict, work–family conflict, and job complexity. Job resource measures included social support, job autonomy, career development opportunities, and distributive justice.

All demand and resource variables were measured using five-point Likert-type scales. Burnout items followed the response format recommended in the underlying burnout instrument.

### 2.4 Reliability and construct validity

Internal consistency reliability was assessed using Cronbach’s alpha. All retained scales demonstrated satisfactory reliability, with Cronbach’s alpha coefficients ranging from 0.819 to 0.925. Reliability and sampling adequacy indicators for the final scales are reported in Table 3.

**TABLE 3:** Reliability and preliminary validity indicators for the final scales.

Construct	Items	Cronbach’s α	KMO
Exhaustion	6	0.925	0.920
Cynicism	3	0.870	0.739
Low professional efficacy	6	0.910	0.887
Workload	3	0.838	0.716
Role conflict	4	0.832	0.805
Work-family conflict	3	0.819	0.712
Job complexity	5	0.859	0.817
Social support	4	0.843	0.809
Job autonomy	6	0.847	0.853
Career development opportunities	3	0.827	0.718
Distributive justice	5	0.865	0.762

Construct validity was evaluated through confirmatory factor analysis (CFA). Standardized factor loadings ranged from 0.692 to 0.878. Composite reliability (CR) values ranged from 0.870 to 0.925, while average variance extracted (AVE) values ranged from 0.634 to 0.691, indicating satisfactory convergent validity.

Discriminant validity was assessed using the Fornell–Larcker criterion. The square roots of AVE exceeded the corresponding inter-construct correlations, supporting adequate discriminant validity. The CFA model fit indices are reported in Table 4.

**TABLE 4:** Confirmatory factor analysis model fit indices.

Model	χ ^2^/df	RMSEA	GFI	CFI	TLI	IFI	Interpretation
Burnout	2.255	0.068	0.911	0.961	0.953	0.961	Good fit
Job demands	2.639	0.072	0.904	0.931	0.913	0.931	Good fit
Job resources	2.612	0.070	0.885	0.906	0.888	0.907	Acceptable fit

### 2.5 Statistical analysis

Descriptive statistics were first calculated for all study variables. Pearson correlation analysis was then conducted to examine bivariate relationships between job demands, job resources, and burnout.

Hierarchical regression analyses were subsequently performed. Demographic variables were entered as control variables in the first step, followed by job demand and job resource variables in subsequent models. This procedure allowed assessment of the incremental explanatory power of the substantive predictors beyond demographic influences.

### 2.6 Ethics statement

This study was conducted in accordance with relevant ethical principles for social science research. The survey was anonymous and participation was entirely voluntary. Respondents were informed of the purpose of the study, the confidential handling of data, and their right to discontinue participation at any stage. Completion and submission of the questionnaire were regarded as implied informed consent. No personally identifiable information was collected.

## 3 Results

### 3.1 Burnout profile

**FIGURE 2:**
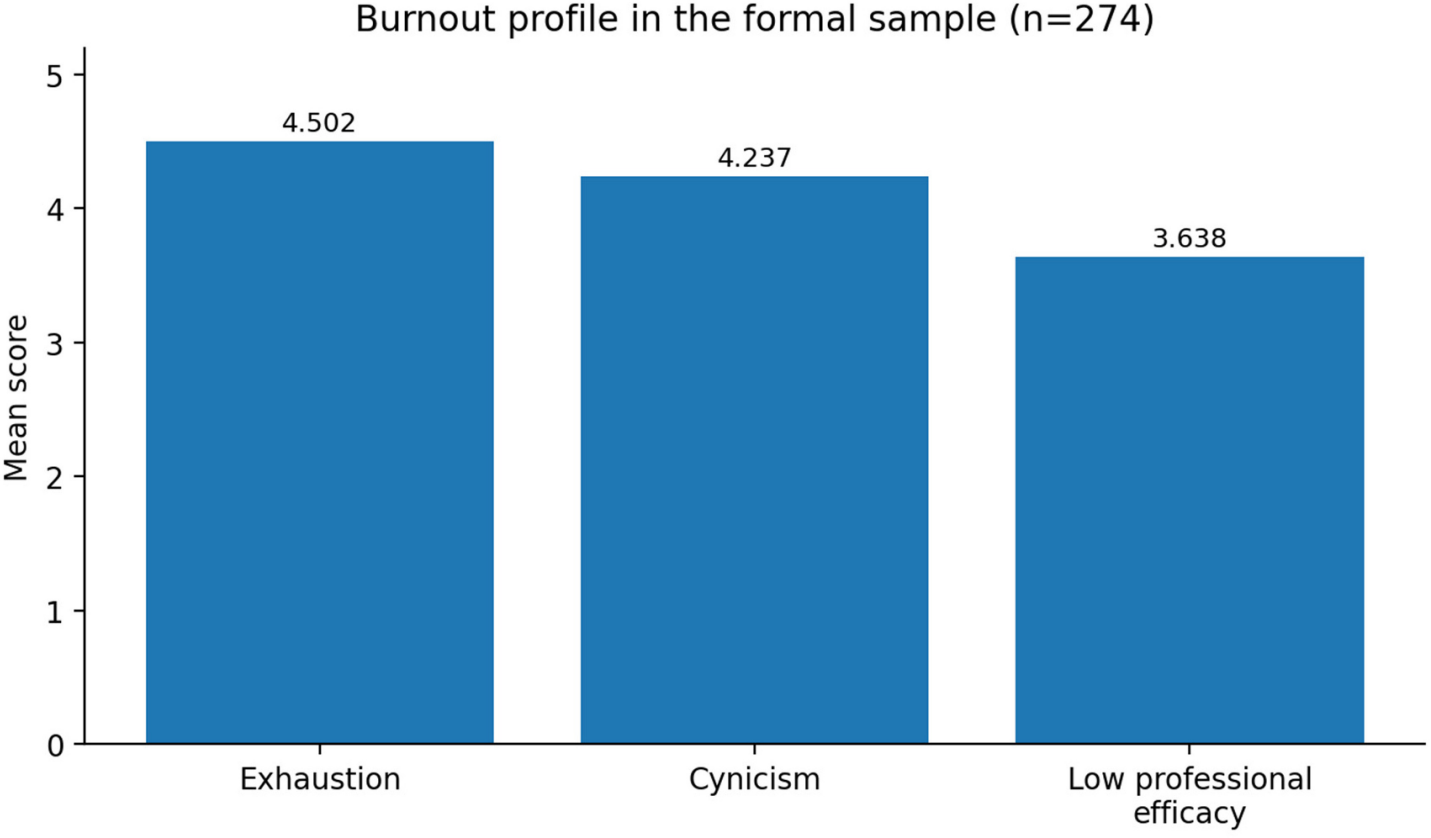
Mean scores for the three burnout dimensions in the formal sample.

The descriptive results indicated relatively elevated burnout scores within the present sample. The mean scores for exhaustion, cynicism, and reduced professional efficacy were 4.502, 4.237, and 3.638, respectively. The dimension- specific mean scores are shown in Figure 2. Exhaustion and cynicism were more prominent than reduced professional efficacy.

These findings suggest that burnout among metro site management personnel is primarily characterized by prolonged energy depletion and psychological detachment from work. However, because no validated occupational cutoff values currently exist for metro site management personnel, these findings should be interpreted as relative indicators rather than clinical classifications of burnout severity.

### 3.2 Reliability and measurement validity

All study constructs demonstrated satisfactory internal consistency reliability. Cronbach’s alpha values ranged from 0.819 to 0.925, exceeding recommended thresholds.

Confirmatory factor analysis provided additional evidence of construct validity. The burnout model demonstrated good fit (χ^2^/df = 2.255, RMSEA = 0.068, CFI = 0.961, TLI = 0.953). The job-demand model also demonstrated satisfactory fit (χ^2^/df = 2.639, RMSEA = 0.072, CFI = 0.931, TLI = 0.913). The job-resource model demonstrated acceptable fit (χ^2^/df = 2.612, RMSEA = 0.070, CFI = 0.906, TLI = 0.888).

Convergent validity was supported by standardized factor loadings ranging from 0.692 to 0.878, composite reliability (CR) values ranging from 0.870 to 0.925, and average variance extracted (AVE) values ranging from 0.634 to 0.691.

Discriminant validity was evaluated using the Fornell–Larcker criterion. For all constructs, the square root of AVE exceeded the corresponding inter-construct correlations, indicating satisfactory discriminant validity.

### 3.3 Correlation analysis

At the aggregate level, job demands were positively associated with burnout (*r* = 0.583, *p* < 0.01), whereas job resources were negatively associated with burnout (*r* = −0.630, *p* < 0.01).

Among the demand dimensions, workload demonstrated the strongest positive association with burnout (*r* = 0.636), followed by role conflict (*r* = 0.434), work–family conflict (*r* = 0.361), and job complexity (*r* = 0.346).

Among the resource dimensions, distributive justice demonstrated the strongest negative association with burnout (*r* = −0.478), followed by social support (*r* = −0.456), career development opportunities (*r* = −0.437), and job autonomy (*r* = −0.330). The full correlation results are presented in Table 5.

**TABLE 5:** Correlations of job demands and job resources with burnout.

Predictor	Exhaustion	Cynicism	Low professional efficacy	Burnout
Workload	0.425**	0.621**	0.470**	0.636**
Role conflict	0.239**	0.443**	0.352**	0.434**
Work-family conflict	0.240**	0.386**	0.232**	0.361**
Job complexity	0.192**	0.380**	0.250**	0.346**
Overall job demands	0.362**	0.599**	0.426**	0.583**
Social support	−0.362**	−0.408**	−0.322**	−0.456**
Job autonomy	−0.214**	−0.298**	−0.278**	−0.330**
Career development opportunities	−0.313**	−0.409**	−0.322**	−0.437**
Distributive justice	−0.317**	−0.432**	−0.395**	−0.478**
Overall job resources	−0.448**	−0.574**	−0.486**	−0.630**

**p < 0.01 (two-tailed).

### 3.4 Hierarchical regression analysis

Hierarchical regression analyses were conducted after controlling for demographic characteristics.

The overall job-demand model indicated that job demands were positively associated with burnout (β = 0.560, *p* < 0.001), explaining 37.3% of the variance in burnout.

The overall job-resource model indicated that job resources were negatively associated with burnout (β = −0.606, *p* < 0.001), explaining 43.7% of the variance.

At the dimension level, workload exhibited the strongest positive association with burnout (β = 0.608), followed by role conflict (β = 0.411), job complexity (β = 0.349), and work–family conflict (β = 0.335).

**FIGURE 3:**
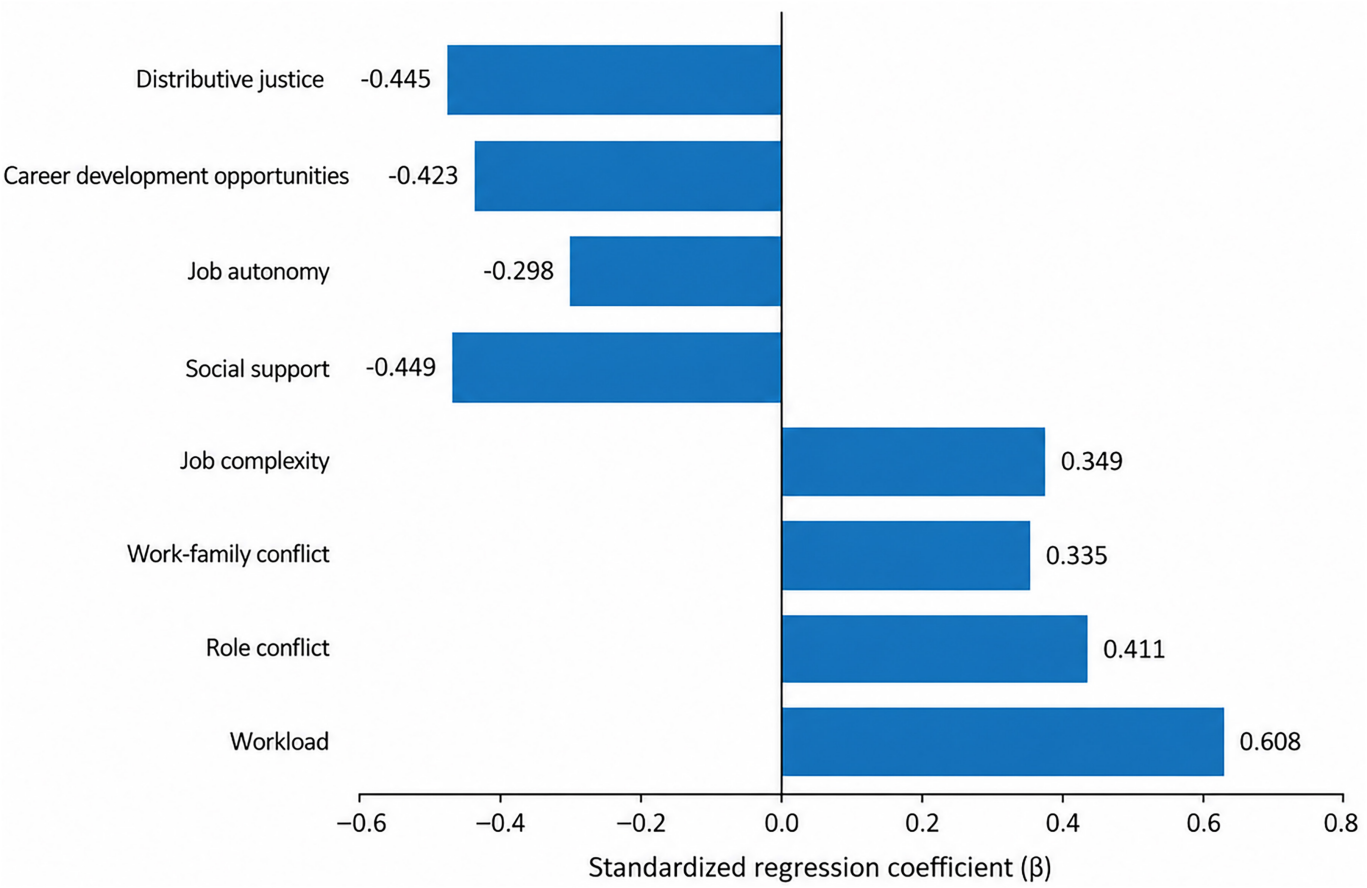
Standardized regression coefficients from the separate dimension-level regression models predicting burnout.

Among job resources, social support (β = −0.449), distributive justice (β = −0.445), and career development opportunities (β = −0.423) demonstrated comparatively strong negative associations with burnout, whereas job autonomy also showed a significant but relatively weaker association (β = −0.298). Detailed aggregate and dimension-level regression results are reported in Table 6. The standardized coefficients from the separate dimension-level models are shown in Figure 3.

**TABLE 6:** Aggregate and dimension-level regression results for burnout.

Model	Predictor	β	*P*	*R* ^2^	Δ *R*^2^	Hypothesis
Model 2	Overall job demands	0.560	<0.001	0.373	0.352	Consistent with H1
Model 3	Workload	0.608	<0.001	0.427	0.407	Consistent with H1a
Model 4	Role conflict	0.411	<0.001	0.247	0.221	Consistent with H1b
Model 5	Work-family conflict	0.335	<0.001	0.182	0.154	Consistent with H1c
Model 6	Job complexity	0.349	<0.001	0.207	0.180	Consistent with H1d
Model 7	Overall job resources	−0.606	<0.001	0.437	0.417	Consistent with H2
Model 8	Social support	−0.449	<0.001	0.283	0.258	Consistent with H2a
Model 9	Job autonomy	−0.298	<0.001	0.173	0.144	Consistent with H2b
Model 10	Career development opportunities	−0.423	<0.001	0.254	0.229	Consistent with H2c
Model 11	Distributive justice	−0.445	<0.001	0.278	0.254	Consistent with H2d

## 4 Discussion

### 4.1 Main findings

This study examined the relationships between job demands, job resources, and burnout among metro site management personnel within the framework of the JD-R model.

The findings indicate that burnout is meaningfully associated with both job demands and job resources in metro construction projects. Consistent with JD-R theory, higher levels of job demands were associated with higher burnout levels, whereas greater availability of job resources was associated with lower burnout levels.

A particularly noteworthy finding concerns the dominant role of workload. Compared with other demand dimensions, workload exhibited the strongest positive association with burnout. This finding may reflect the organizational realities of metro construction projects, where compressed schedules, overlapping work tasks, prolonged overtime, and strict milestone requirements place substantial demands on site management personnel.

Role conflict, work-family conflict, and job complexity also demonstrated significant associations with burnout. Metro site management personnel frequently operate across organizational boundaries, coordinate multiple stakeholders, and respond to changing technical and environmental conditions. These characteristics may increase uncertainty and contribute to psychological strain.

On the resource side, social support, distributive justice, and career development opportunities demonstrated particularly strong protective associations. The highly interdependent nature of metro project work may explain the importance of these factors. Supportive relationships, fair treatment, and opportunities for professional growth may help employees maintain motivation and cope more effectively with demanding work environments.

### 4.2 Theoretical implications

The present study extends the JD-R literature in several important ways.

First, it demonstrates the applicability of the JD-R model within a temporary, safety-critical, and coordination-intensive project environment represented by metro construction projects. Compared with occupations commonly examined in burnout research, metro site management personnel operate within a distinctive organizational context characterized by multiple stakeholder interfaces, complex coordination demands, and substantial safety responsibilities.

Second, the findings indicate that the internal composition of job demands and job resources is theoretically meaningful. Not all demand and resource dimensions contribute equally to burnout. Workload showed the largest standardized coefficient among the separate regression models. This finding suggests that future JD-R research should pay greater attention to the relative salience of specific dimensions rather than treating demands and resources as homogeneous categories.

Third, the study contributes to project management research by conceptualizing burnout as a project governance issue embedded in work design, resource allocation, and organizational support systems. Burnout should therefore be understood not only as an individual psychological outcome but also as an organizational challenge with implications for project effectiveness and workforce sustainability.

### 4.3 Practical implications

The findings provide several practical implications for metro construction organizations.

First, workload management should be treated as a strategic priority for burnout prevention. Among all job-demand dimensions, workload demonstrated the strongest association with burnout. Organizations should therefore pay particular attention to staffing allocation, scheduling practices, overtime management, and resource coordination during critical project phases. More realistic project planning and improved workforce deployment may help reduce excessive strain on site management personnel.

Second, organizations should strengthen social and organizational resources. The strong associations observed for social support and distributive justice suggest that supportive supervisory relationships, collaborative team environments, and fair reward systems represent important protective mechanisms against burnout. Management practices that promote communication, trust, and organizational fairness may therefore contribute to improved employee wellbeing.

Third, career development opportunities should receive greater managerial attention. Metro site management personnel frequently face demanding work conditions and long project cycles. Providing structured training programs, transparent promotion pathways, and opportunities for professional growth may enhance employee motivation and reduce burnout risk.

Finally, burnout prevention should be integrated into broader project governance systems. Rather than treating burnout solely as an individual psychological issue, organizations should recognize its potential implications for safety management, communication effectiveness, workforce retention, and project performance. Human-centered management approaches may therefore contribute to both employee wellbeing and organizational sustainability.

### 4.4 Strengths and limitations

This study possesses several strengths.

First, the study was grounded in a well-established theoretical framework and applied the JD-R model to an underexplored occupational context. Second, the research adopted a two-stage survey design involving both pilot and formal investigations, which enhanced the quality of the measurement instrument. Third, the study evaluated construct validity through both exploratory and confirmatory procedures, providing additional confidence in the measurement results.

Several limitations should also be acknowledged.

First, the sample was drawn primarily from construction-side participants involved in metro projects and therefore may not fully represent all stakeholders within the metro project ecosystem, such as owners, design organizations, and supervision agencies. Future studies may benefit from broader stakeholder coverage.

Second, the cross-sectional research design limits causal inference. Although the observed relationships are consistent with JD-R theory, the findings should be interpreted as associations rather than definitive causal effects. Longitudinal research designs would provide stronger evidence regarding temporal relationships among job demands, job resources, and burnout.

Third, because all variables were collected through self-report questionnaires, common method variance cannot be entirely excluded. Although anonymity, confidentiality, and voluntary participation were used to reduce potential bias, future studies may benefit from multi-source or multi-wave data collection approaches.

Finally, other potentially relevant variables, including psychological capital, resilience, leadership style, and safety climate, were not incorporated into the present model. Future research should investigate how these factors interact with job demands and job resources in shaping burnout among construction management personnel.

## 5 Conclusion

Drawing on the Job Demands–Resources (JD-R) framework, this study examined the relationships between job demands, job resources, and burnout among metro site management personnel in China.

The findings indicate that workload, role conflict, work–family conflict, and job complexity were positively associated with burnout, whereas social support, job autonomy, career development opportunities, and distributive justice were negatively associated with burnout. Among these factors, workload showed the largest standardized coefficient among the separate regression models, while social support, distributive justice, and career development opportunities demonstrated the most influential protective associations.

The study contributes to the literature by extending the JD-R model to a temporary, safety-critical, and coordination-intensive project environment represented by metro construction projects. The findings further demonstrate that specific demand and resource dimensions differ substantially in their associations with burnout and therefore should not be treated as interchangeable components within the JD-R framework.

From a practical perspective, the results highlight the importance of workload management, organizational support systems, distributive fairness, and career development opportunities in reducing burnout among metro site management personnel.

Although the cross-sectional design precludes strong causal conclusions, the study provides useful evidence for understanding burnout in project-based organizations and offers practical guidance for promoting employee wellbeing and organizational sustainability in large infrastructure projects.

## Data Availability

The original contributions presented in this study are publicly available. This data can be found here: https://doi.org/10.5281/zenodo.21153010.

## Appendix

Final measurement items used in the formal survey.

**TABLE A1:** Constructs, item codes, and representative item wording.

Construct	Item codes	Representative wording/note
Burnout-exhaustion	EX1–EX6	Examples: “Project management work makes me physically and mentally exhausted”; “I feel drained after work.”
Burnout-cynicism	CY1–CY3	Examples: “I increasingly doubt the meaning of my work;” “I am less engaged in project management work than before.”
Burnout-low professional efficacy	LP1–LP6	Examples: “I feel that I can solve difficult project problems”; reverse-coded to reflect reduced professional efficacy.
Workload	WL1–WL3	Examples: “I often do not have enough time to complete my work”; “I frequently work overtime.”
Role conflict	JC1–JC4	Examples: “I have to perform tasks outside my responsibilities”; “I work with multiple groups that operate differently.”
Work-family conflict	GJ1–GJ3	Examples: “My work occupies too much time with my family”; “Work prevents me from fulfilling family responsibilities.”
Job complexity	GF1–GF5	Examples: “I need to coordinate relationships with different departments”; “My work procedures are not clearly defined.”
Social support	SZ1–SZ4	Examples: “I can obtain help from supervisors when I encounter difficulties”; “My colleagues are willing to help me.”
Job autonomy	GZ1–GZ6	Examples: “I can decide how to complete my work”; “I have sufficient voice in my work.”
Career development opportunities	ZF1–ZF3	Examples: “I have opportunities to participate in training”; “My job has development prospects.”
Distributive justice	ZG1–ZG5	Examples: “My compensation is fair relative to colleagues”; “My compensation reflects my contributions.”
